# Joint Item Response Models for Manual and Automatic Scores on Open-Ended Test Items

**DOI:** 10.1017/psy.2025.10018

**Published:** 2025-06-16

**Authors:** Daniel Bengs, Ulf Brefeld, Ulf Kroehne, Fabian Zehner

**Affiliations:** 1 Leibniz Institute for Research and Information in Education, Frankfurt, Germany; 2 Leuphana University Lüneburg, Lüneburg, Germany; 3 Centre for International Student Assessment

**Keywords:** automatic scoring, item response modeling, large-scale assessment

## Abstract

Test items using open-ended response formats can increase an instrument’s construct validity. However, traditionally, their application in educational testing requires human coders to score the responses. Manual scoring not only increases operational costs but also prohibits the use of evidence from open-ended items to inform routing decisions in adaptive designs. Using machine learning and natural language processing, automatic scoring provides classifiers that can instantly assign scores to text responses. Although optimized for agreement with manual scores, automatic scoring is not perfectly accurate and introduces an additional source of error into the response process, leading to a misspecification of the measurement model used with the manual score. We propose two joint models for manual and automatic scores of automatically scored open-ended items. Our models extend a given model from Item Response Theory for the manual scores by a component for the automatic scores, accounting for classification errors. The models were evaluated using data from the Programme for International Student Assessment (2012) and simulated data, demonstrating their capacity to mitigate the impact of classification errors on ability estimation compared to a baseline that disregards classification errors.

Open-ended response formats (i.e., constructed-response items) can increase an instrument’s construct validity (Ihme et al., 2017; Lim, 2019); however, traditional applications in educational testing require the provision of human coders to manually score responses, which is time-consuming and entails substantial costs.

Moreover, manual scores are not available during the testing. Hence, the evidence enclosed in responses to open-ended items is not available for the immediate scoring and feedback of linear tests, and it also does not contribute to interim ability estimation and routing decisions in adaptive designs, such as computerized adaptive testing (CAT; Weiss 1982) or multi-stage adaptive testing (MSAT; Yan et al., 2014).

The automatic scoring of text responses significantly reduces the workload required for manual scoring. For scoring, categories (i.e., codes or scores) are algorithmically assigned to textual response content (Bauer and Zapata-Rivera, 2020), and in automatic scoring, also referred to as automatic short answer grading (e.g., Burrows et al., 2015), this is done by computers. The idea of programming computers to evaluate the quality of students’ textual work products (Foltz et al., 2020) can be traced back to the 1960s in the context of automatic essay scoring (Page, 1966). The growing demand for open-ended response formats in large-scale assessments in the 1990s (Bennett 1993) led to a new focus on automatically scoring short responses (Bejar, 1991; Kaplan, 1991).

Coupled with advances in the underlying methodology (see Burrows et al., 2015, for a historical outline), tremendous progress has been made since the inception of the field, particularly with the introduction of pretrained large language models with transformer architectures, such as bidirectional encoder representations from transformers (BERT; Devlin et al., 2019). Predominantly, supervised learning methods are used, in which manual scores serve to label the training data and hence define the ground truth that the classifier is optimized to reproduce. However, a variety of automatic scoring paradigms have emerged over time. The initial development of the scoring model is a fundamental characteristic of these methods. This model comprises rules that map features to scores, and for evaluation and production, it is subsequently applied to score new responses (Williamson et al., 2012).

Building on Zesch et al. (2023), roughly four paradigms can be distinguished: 1) models hand-crafted by assessment or domain experts using, for example, regular expressions (Cai et al., 2019); 2) models trained by semi-supervised machine learning, which teams up human and machine (Andersen et al., 2023; Wolska et al., 2014); 3) models trained by supervised learning with traditional machine learning (Sakaguchi et al., 2015); and 4) pre-trained deep learning models with transformer architecture that can be fine-tuned (i.e., optimized) to the scoring task at hand (e.g., Bonthu et al., 2021; Camus and Filighera, 2020; Haller et al., 2022). Common feature sets that form the input or central representations of models are *n*-grams (Higgins et al., 2014) or, more commonly, embeddings that represent semantics (Zehner et al., 2016). The underlying methodologies further differ in other characteristics, such as the explainability (i.e., transparency) of the resulting classifications. Improved explainability is usually associated with more traditional learning algorithms, such as rule-based learning or clustering; however, new approaches have recently emerged to create a certain degree of post hoc explainability for deep neural nets (Chefer et al., 2021; Gombert et al., 2023; Lottridge et al., 2023).

Regardless of the underlying paradigm or feature set employed, all methods follow the basic concept of text classification, which may result in false-positive or false-negative classifications. The integration of these false classifications into an appropriate measurement model constitutes the core of this study. Johnson et al. (2022) posited that automatic scoring models should be optimized for the true value, represented by the mean of multiple human ratings, rather than the observed human ratings, as this would be more optimal. However, because of operational constraints, international large-scale assessments only apply double coding to a limited set of responses to monitor interrater reliability, limiting the practical applicability of this approach.

Despite the methodological advances, the automatic scoring of open-ended test items remains challenging, and automatic scores are generally not perfectly accurate. This implies that automatic scoring introduces an additional source of error, and hence uncertainty, into the process that generates the observable response. As the measurement model for manual scores disregards potential errors arising from automatic scoring, it cannot be applied directly to automatic scores. Therefore, the application of automatic scoring in adaptive test formats faces a dilemma: While a model for manual scoring is available during online testing, the actual scores are not. Simultaneously, automatic scores can be obtained; however, without a corresponding measurement model, they are not immediately available for measurement.

In computer-based assessment practices, this dilemma has been addressed by avoiding reliance on open-ended items for online scoring and adaptivity. In the MSAT framework, this is made feasible by combining open-ended and closed item formats in each module. The provisional ability estimates used for routing decisions rely solely on closed-format items that can be scored immediately, whereas manual scores for open-ended items contribute to the final ability estimate used for reporting when they become available. This approach featured prominently in the 2018 Programme for International Student Assessment (PISA), as detailed by Yamamoto et al. (2018). Despite reconciling the use of open-ended items with, to some extent, the increased measurement efficiency afforded by adaptive testing, this approach has obvious drawbacks. First, the required balancing of open-ended and closed item formats imposes additional requirements and constraints on item development and test assembly. Second, routing decisions are made without taking advantage of information in response to open-ended items. Third, the approach transfers neither to item-level adaptivity in computerized adaptive testing (CAT) nor to the automatic scoring of linear tests. Finally, human coders are required during operational testing.

Motivated by these issues, we investigate joint models for manual and automatic scores. Our modeling approach aligns with the role of the manual score as the ground-truth label during supervised learning. In other words, we regard the manual score as the gold standard, which is reproduced, albeit imperfectly, by an automatic scoring algorithm. Consequently, the discrepancies between the manual and automatic scores, which we regard as classification errors, are a primary focus of our investigation.

In this study, we propose and empirically test two structurally different joint models for manual and automatic scores. We assume that manual scores follow a given IRT model. The model for the manual scores is then extended by a component that captures the classification error and posits a generative process for the automatic scores.

By marginalizing out the manual score, we derive measurement models that allow for inference of the latent trait using only the automatic score while accounting for classification errors. We show that these marginal models are closely related to the well-known four-parameter family of IRT models (Barton & Lord, 1981) and their generalizations. The use of our marginal models enables automatic scoring when immediate updates of provisional ability estimates are required, as in computer-based adaptive tests.

The remainder of this paper is organized as follows. We first formulate the proposed models, discuss the estimation of model parameters, and derive marginal models for automatic scores. Subsequently, we report the results of a simulation study that assesses parameter recovery. The simulations also investigate how ability estimates are affected if classification errors are ignored; that is, the model for the manual score is used to analyze automatic scores affected by different levels of classification error. We then present an empirical example using data from eight open-ended items from the PISA 2012 (OECD, 2014) reading domain and two different classifiers for each item to provide automatic scoring, in which we apply marginal models for ability estimation based on automatic scores and evaluate reliability relative to ability estimation based on manual scores. Finally, we discuss the results and their implications and indicate directions for further research.

## 1. Joint item response models for manual and automatic scores

Our core modeling assumption is that the automatic score is related to the manual score through an error-prone process that can be described by modeling the classification error probabilities conditional on the manual score. The first two subsections introduce notation and present the formulation of the proposed models. The third subsection discusses parameter estimation, whereas the fourth subsection derives a marginal measurement model that depends only on automatic scores. Finally, we discuss the special case of dichotomous items relevant to the empirical example.

### 1.1. Notation

Throughout the paper, we index a set of individuals by 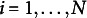 and a set of open-ended test items by 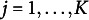 . Let 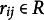 denote raw text responses to the test items where 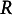 denotes the universe of possible text responses. Let 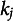 denote the number of response categories of item 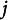 and let 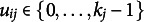 denote the manual score, that is, an ordinal score assigned to each *r_ij_* by a human coder. We assume that the latent trait (i.e., ability) that the instrument is designed to measure and the manual scores are related by a one-dimensional IRT model. More specifically, we assume that the manual scores are realizations of a random variable 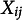 such that the probability of observing a manual score in category *u* is given by
(1)

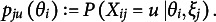



Here, 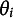 denotes individual 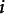 *’*s ability and 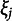 denotes the vector of item parameters of item 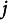 , which controls the shape of the item characteristic curve (ICC) and, after sufficiently accurate calibration, is assumed to be known for each item.

Automatic scoring for some item 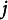 is a mapping

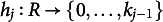

 which assigns an automatic score to any text response in 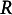 ; that is, 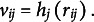 We assume a supervised learning approach that optimizes 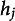 to maximize agreement with the manual score by training the classifier for item 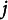 on data 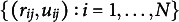 . We write the automatic scores 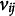 as realizations of random variables 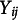 , whose conditional distributions are parametrized in terms of classifier parameters 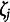 . Finally, it will be convenient to use vector and matrix quantities 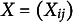 , 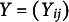 , 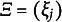 , 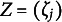 , 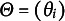 , 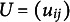 , and 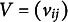 .

### 1.2. Model formulation

Let
(2)

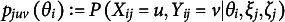



denote the joint probability of observing a manual score 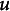 and an automatic score 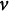 to item 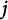 for individual 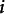 , given the individual’s ability 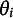 , item parameters 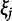 , and classifier parameters 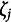 .

By the definition of conditional probability, we may write
(3)

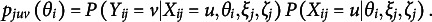



We assume that the manual score for item 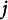 is conditionally independent of classifier parameters 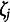 given item parameters 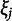 and ability 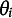 , and hence, its factor in Equation (3) takes the form
(4)

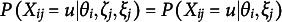



(5)

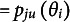

 of the IRT model for the manual score in Equation (1). Similarly, we assume that the automatic score is conditionally independent of item parameters 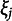 , given ability 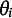 and classifier parameters 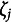 , allowing us to write its factor in Equation (3) as
(6)

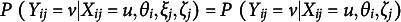



(7)

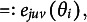

arriving at
(8)

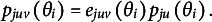



As the conditional probability distribution 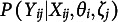 is determined by the error probabilities 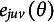 , 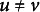 , the factor for the classifier can essentially be regarded as a model of classifier error rates. As per our assumptions, the classifier error rates 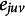 can vary with the ability level, we call the resulting joint model for the manual and automatic scores in Equation (8) the variable error rate (VER) model. A simpler special case of the VER model results if we make the additional assumption that the automatic score is conditionally independent of ability, that is,
(9)

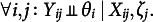



Then, the conditional probabilities 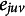 governing the classifier model in the VER model do not depend on 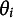 and hence, it holds that
(10)

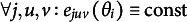

and we may drop the dependency on 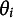 in the error rates model. The resulting joint model
(11)

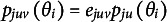

 for the manual and automatic scores then includes only constant classifier error rates; hence, it is referred to as the constant error rate (CER) model.

### 1.3. Parameter estimation

We consider the problem of estimating the classifier parameters *Z* when given the observed data *U* for manual scores and *V* for automatic scores.

In the following, we first address the CER Model. We have that
(12)

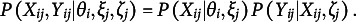



Hence, under standard assumptions, the log-likelihood function is given by
(13)

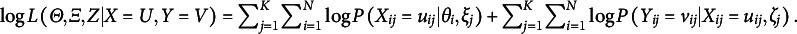



The double sum on the left is the log-likelihood of the IRT model for the manual score, whereas the double sum on the right pertains to the classifier error model. The terms relating to the classifier error model do not include a dependency on person and item parameters; therefore, the sums in Equation (13) can be maximized independently to obtain the maximum likelihood estimates of the parameters of the joint model.

The right-hand double sum in Equation (13) decomposes further into terms depending only on one 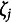 each, and, hence, can be maximized for each item separately. With the model for 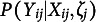 being categorical, the classifier parameters are formed by fixed probabilities of each error type for each item. That is,

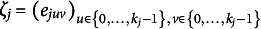

 and the maximum likelihood estimates are given (Koller and Friedman, 2010, p. 726) by
(14)

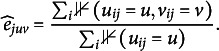



In particular, if item 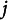 is dichotomous, then the maximum likelihood estimates are determined by 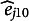 and 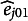 . In the context of binary classification, 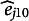 is the false-negative rate of the classifier, defined as the number of training instances (responses to item 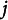 ) falsely classified as incorrect divided by the total number of correct responses. Analogously, 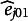 is the false-positive rate of the classifier, defined as the number of training instances falsely classified as correct divided by the total number of incorrect responses. Again, the manual scores serve as the ground truth. The complementary probabilities 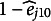 and 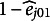 are the classifier sensitivity and specificity, respectively.

The decomposition and separate estimability of the CER model parameters make it possible to calculate maximum likelihood parameter estimates by combining maximum likelihood estimates of person and item parameters, 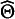 and 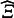 , obtained from the calibration of the model for the manual scores, with maximum likelihood estimates of the classifier parameters, 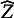 , which can be independently estimated per item. This also implies that the person parameters obtained by calibrating the CER model are necessarily on the same scale as those obtained by calibrating the IRT model for the manual score.

In the case of the VER model, the direct dependency of the automatic score on ability results in a possible divergence of the scales when simultaneously estimating the person, item, and classifier parameters from scores 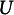 and 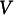 . Hence, the scales need to be linked. We propose linking using a fixed-parameter approach. That is, when calibrating the VER model, we regard the classifier parameters 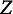 as the parameters of interest, while person and item parameters are nuisance parameters that are fixed to point estimates 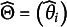 and 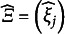 , obtained from the calibration of the IRT model for the manual scores. This approach also simplifies fitting classifier models that capture the 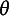 -dependency of the error rates. Thus, the log-likelihood function becomes:

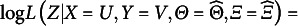



(15)

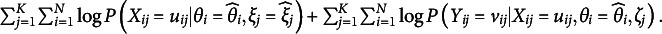



Because the first sum is constant, only the second sum is maximized. The second sum is decoupled into separate terms for each item. It is maximized by finding the maximizing 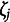 for each item 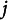 . The actual estimation of each classifier parameter 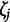 then depends on the parametric form chosen for the probability model for the classifier error rates. In the case of dichotomous items discussed in greater detail below, we assume logit models for the VER error probabilities, which, using point estimates for ability, become manifest logistic regressions. To evaluate the viability of our approach for fitting VER classifier parameters, we conducted a simulation study, as described below.

### 1.4. Measuring the latent trait using automatic scores

In this section, we consider the measurement of an individual’s abilities during testing. To simplify the notation, we drop the subject index 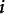 . We may assume item and classifier parameters 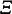 and 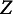 as given, as well as a vector of automatically-coded responses 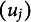 , while the manual scores are not observed. To facilitate inference on 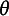 in this scenario, we derive an expression for 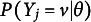 , the probability of observing the automatic score in terms of the latent trait, using the law of total probability as follows:
(16)

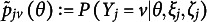



(17)

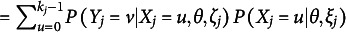



(18)

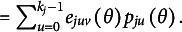



As the manual score 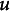 is marginalized out in the expression for 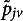 , its observation is not a prerequisite for inference on 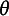 based on Equation (18). Hence, Equation (18) provides a measurement model for 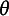 based only on the automatic score 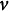 . Note that the above derivation generalizes the decomposition of the three- and four-parameter IRT models, respectively, used by Béguin and Glas (2001) and Culpepper (2016) in the context of MCMC estimation for the normal ogive variant of these models. In the cited works, 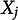 is an auxiliary augmented variable whose role is entirely technical. In our case, 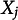 has a substantive interpretation and is, in principle, empirically observable as a manual score.

The actual form of the measurement model in Equations (16)–(18) depends on the following two factors: The first is the question of whether constant or varying classifier error rates are used, and if applicable, how the dependency on ability is modeled. The second factor is the parametric form of the IRT model for 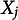 , which has not yet been specified. We address the former aspect in the context of our empirical study and turn to the latter in the next section, discussing the case of a dichotomous response model of the 4PL family.

### 1.5. Application to dichotomous items

Although our modeling framework encompasses polytomous items, in the remainder of the manuscript, we limit the discussion to dichotomous items, which are of special interest for our empirical study. In this section, we complete the specification of the measurement model in Equation (18) assuming that the model underlying the manual score is the 4PL model. The assumption of dichotomy allows us to simplify the notation as we only need to specify the probability of a response scored as correct (coded as 1) and may drop the index for the response category. We define the ICC of the 2PL as
(19)

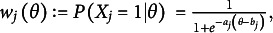

 and may then write the ICC of the 4PL (Barton & Lord, 1981) as
(20)

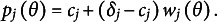



Parameters 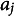 and 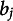 of the 2PL model are referred to as the *discrimination* and *difficulty* parameters of item 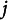 . The additional parameters 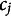 and 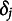 introduced in the 4PL are asymptotic parameters; 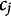 is referred to as the *guessing* parameter, and 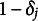 as the *slipping* parameter.

The conditional probabilities of inaccurate response classification can be represented by
(21)

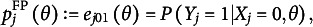

 the conditional probability of false-positive classification, and
(22)

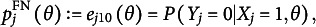

 the conditional probability of false-negative classification.

By writing the expression for 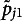 from Equation (18) in terms of 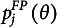 and 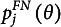 and simplifying, we get
(23)

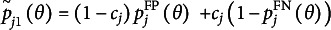




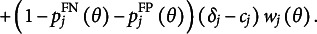



Letting
(24)

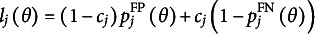



and
(25)

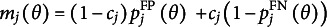




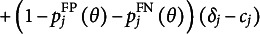



Equation (23) can be written in close similarity to the 4PL model as
(26)

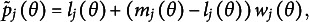

 where 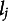 takes a technically similar role as the third parameter of the 4PL model, and 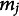 plays a similar role to the fourth parameter. Here, 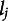 and 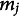 are functions of 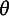 . Hence, the model in Equation (26) generalizes the 4PL model and is referred to as the generalized 4PL (G4PL) model. From the G4PL, the usual 4PL model is recovered if conditional independence, as per Equation (10), holds, and 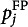 and 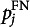 are constant. A nested special case arises if the model for the manual score is a 2PL model (i.e., 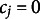 and 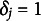 ). Then, the marginal model for the automatic score is a 4PL model, where the third parameter is given by 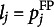 and the fourth parameter is given by 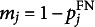 , that is,
(27)

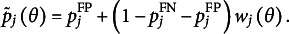



As a practical consequence of the considerations above, statistical routines for the 4PL IRT model, which are implemented in common software packages, such as SIRT (Robitzsch, 2024) and PP (Reif & Steinfeld, 2021), can be applied to estimate person parameters from automatic scores under the CER model.

As a parametric form of the probabilities in Equations (21) and (22) in the VER model, we use the logit models
(28)

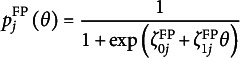

 and
(29)

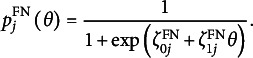



When fitting the VER model using point estimates for ability as proposed (Equation (15)), the models in Equations (28) and (29) become manifest logistic regression models that are fitted for each item and each error type. The maximum likelihood estimates of the fixed error probabilities in the CER model are given in Equation (14). The CER model can also be regarded as a special case of Equations (28) and (29), where only the intercept is fitted, resulting in an equivalent parameterization of the classifier parameters of the CER model on the logit scale.

## 2. Simulation study

A simulation study was conducted to investigate the parameter recovery of the CER and VER models. In the simulation, we also studied the effect of ignoring classification errors. To this end, we estimated person parameters from automatic scores using the 2PL model that generated the manual scores, while the model generating the automatic scores was either the CER or VER model. All R scripts required to reproduce the simulation results are available at OSF^1^.

### 2.1. Data generation

For each of the two models (CER and VER), we generated 100 datasets for 4 × 4 × 3 conditions: four different numbers of items ( 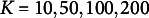 *)* crossed with four different sample sizes ( 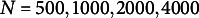 ) crossed with three conditions for the classifier error rates, which varied the balance between the two error types. Person parameters and item difficulties were drawn from 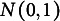 , and item discriminations were drawn from 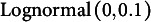 . For the CER model, doubled classification error rates were drawn from Beta 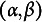 distributions, limiting the range of classification error rates to 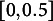 . In the balanced error rates condition, we set 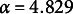 and 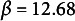 , such that the 2.5 and 97.5 percentiles of error rates were at 0.05 and 0.25, respectively. We defined two conditions with imbalanced error rates by increasing either the false-positive or false-negative rates of the balanced error rate condition. The increased error rates were defined by setting 
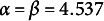
 , such that the 2.5 and 97.5 percentiles of error rates were at 0.1 and 0.4, respectively. For the VER model, the slopes of the error rates were drawn as 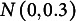 . Manual scores were then sampled from a 2PL model, and automatic scores were generated from the manual scores by introducing classification errors according to the CER or VER model assumptions.

### 2.2. Parameter estimation

We focused on the recovery of abilities and classifier parameters and used the true (data-generating) item difficulties and discriminations in the simulation. We estimated persons’ abilities using manual scores and the 2PL model. The CER classifier model parameters (constant false-positive and false-negative rates) were estimated from manual and automatic scores and reported on a logit scale as error rate intercepts to allow comparison with the VER classifier parameters. The VER classifier parameters (intercepts and slopes of the logistic regression error rate models) were estimated using manual scores, automatic scores, and the 2PL ability estimate derived from manual scores as a point estimate for ability. We then computed the ability estimates for the marginal 4PL and G4PL models using the recovered classifier model parameters, automatic scores, and true item parameters. As a baseline, we estimated abilities using the 2PL model for manual scores but with automatic scores, effectively ignoring the possibility of classification error. All the ability estimates were calculated as expected a posteriori (EAP) estimates, with a prior distribution of 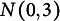 .

### 2.3. Performance measures

For each dataset and parameter group, we computed the bias, root mean square error (RMSE), and Pearson correlation coefficient between the true parameters and their estimates. For a more compact presentation, we did not distinguish between the two error types of the classifier model parameters. The performance measures were averaged across repetitions for each condition.

### 2.4. Results and discussion

**Figure 1:**
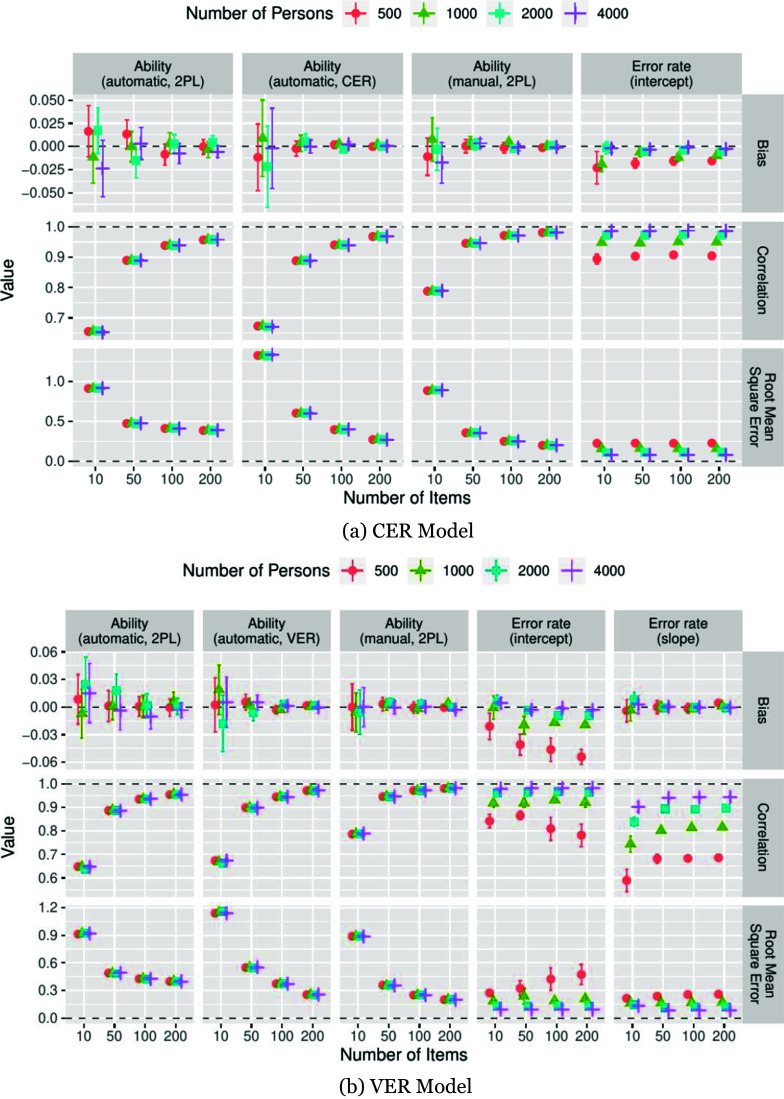
Average performance measures for the CER model (a) and VER model (b) in the balanced error condition. Error bars: 95% confidence intervals.

**Figure 2:**
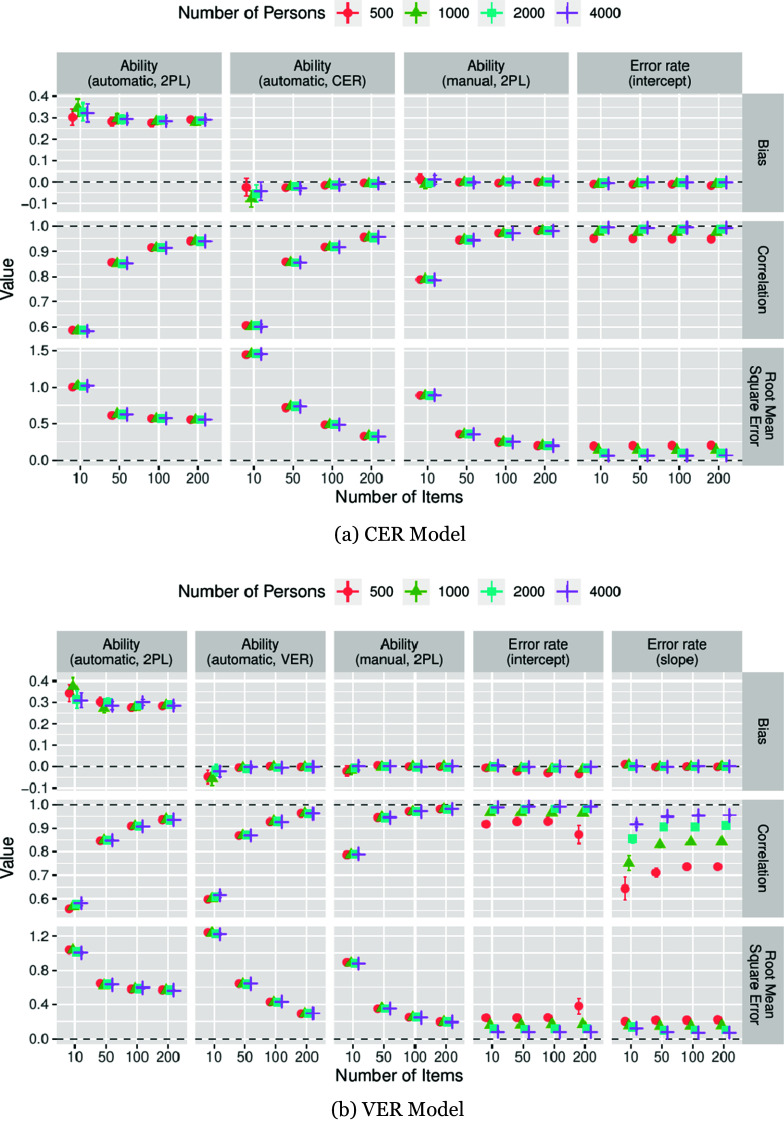
Average performance measures for the CER model (a) and VER model (b) in the unbalanced error condition with increased false-positive rate. Error bars: 95% confidence intervals.

The average performance measures for the balanced error condition and the unbalanced error condition with increased rates of false-positive errors are presented in Figures 1 and 2, along with 95% confidence intervals. The results for the unbalanced error condition with increased false-negative error rates are presented in Figure A1 in the Supplementary Material).

#### 2.4.1. Recovery of ability

In terms of RMSE (Figures 1 and 2, bottom rows, first three panels from the left), the ability parameters were recovered most efficiently from the manual scores when the analysis model matched the model generating the data, namely, the 2PL. This is not surprising, given that these estimates were unaffected by both estimation errors in the model parameter estimates and classification errors or model misspecification. Contrary to our expectations, the RMSE of the ability estimates recovered from the automatic scores using the 2PL model was lower than those of the CER and VER models when the number of items was low (CER: 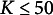 ; VER: 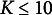 ). As the number of items increased, the RMSE of the ability estimates of the CER and VER models fell below that of the 2PL model (automatic scores) and approached that of the 2PL model (manual scores). Correlations (Figures 1 and 2, middle rows, first three panels from the left) between the true and estimated abilities were the highest for the 2PL model (manual scores), whereas those for the VER and CER models slightly exceeded those of the 2PL model (automatic scores). In the balanced-error conditions, the ability estimates appeared practically unbiased (Figure 1, top rows, first two panels from the left). However, in the unbalanced error condition with an increased false-positive rate, a considerable positive bias (approximately .3 logits) in ability estimates was observed for the 2PL model (automatic scores). Notably, the CER and VER models did not suffer from this marked overestimation of ability but showed a tendency to slightly underestimate ability that diminished when the number of items increased (Figure 2, top rows, first two panels from the left). A complementary pattern emerged for the unbalanced error condition, with increased false-negative rates (Figure A1 in the Supplementary Material).

#### 2.4.2. Classifier model parameters

The estimation of the error-rate intercepts in the CER model proved unproblematic (Figures 1a and 2a, rightmost columns). A very slight underestimation of the error rates could be observed that vanished when the sample sizes were increased.

A similar pattern was observed for the VER model (Figures 1b and 2b, two rightmost columns). Relative to the CER model, the estimation of the variable error rates imposed considerably higher requirements on the sample size. Regarding the estimation of error rate intercepts, for the smallest sample size of 500 persons, all performance measures degraded when the number of items increased but improved to an acceptable level when the sample size increased (Figures 1b and 2b, second column from the right). In contrast to the findings for the CER model, the sign of the mean bias in the error rate intercept estimates was not consistent for the VER model but seemed to depend on the sample size when the number of items was low (Figures 1b and 2b, top row, second panel from the right). Estimates of error rate slopes appeared virtually unbiased on average; however, relatively large sample sizes were required to achieve high correlations with the true parameters (Figures 1b and 2b, middle row, rightmost panel).

These findings suggest that, under conditions analogous to those of our simulation, a sample size of at least 1,000, and preferably more, is necessary to obtain reliable estimates of the VER classifier parameters.

#### 2.4.3. Overall evaluation

Overall, parameter recovery for both models was satisfactory when the sample size was sufficiently large. As a general pattern, the classifier parameter estimation improved when the number of persons increased, and the person parameter estimates improved when the number of items increased. As an exception, for the two smallest sample sizes used, the estimation of the classifier model parameters of the VER model did not improve—or even degraded—when the number of items was increased, indicating that the sample size requirements of the VER model were considerably higher than those of the CER model. The simulations also highlighted the risk of obtaining biased ability estimates when error-prone automatic scores are used with the manual score model. Here, the bias can be attributed to the presence of a greater proportion of false-negative or false-positive automatic scores, which led to an underestimation or overestimation of ability, respectively. This strong and systematic bias did not affect the ability parameters recovered using the CER and VER models, which remained unbiased, except for a slight tendency toward overcompensation when the number of items was low.

## 3. Empirical example: Automatically scored open-ended items in the PISA 2012 (OECD, 2014) reading assessment

In this section, we report on the application of the proposed models to a set of eight items from the PISA 2012 (OECD, 2014) reading assessment. Two automatic scoring algorithms were used for each item. We tested the assumption of conditional independence of classifier errors and ability and fitted the proposed CER and VER models. We discuss the impact of classification errors on item characteristic curves and item information curves under the respective marginal models for automatic scores and report the reliability of ability estimates obtained from the automatic scores using the marginal models relative to ability estimates obtained using the manual scores.

### 3.1. Dataset

We used data from the German PISA 2012 (OECD, 2014) sample (see Prenzel et al., 2013, for a detailed sample description), focusing on eight dichotomous items from the reading assessment. The dataset comprised responses from 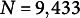 persons. Owing to the incomplete design, the number of responses for each item varied between 4152 and 4234. Based on the distinction between methodological paradigms, two automatic coding methods were chosen to obtain classifiers for the raw text responses. The first classification method (C1) can be considered a more traditional baseline method that uses supervised learning with higher explainability, because it is based on clustering representations of responses in a semantic space constructed by latent semantic analysis (Deerwester et al., 1990). The scores for this method were obtained from Zehner et al. (2016). The second classification method (C2) stems from the family of modern transformer models. It was implemented by the present authors using a pre-trained deep learning model called German Uncased ELECTRA (Reissel & May, 2020) as the basis for fine-tuning a neural network classifier. The resulting dataset thus comprised manual scores for eight items along with one set of automatic scores for each of the two classifiers, C1 and C2, yielding 16 automatically scored items. We labeled the automatically scored items by concatenating the item and classifier labels, separated by a slash, such that, for example, R455Q03/C1 references item R455Q03, scored with C1.

Both classification methods exhibited good to excellent performance with respect to agreement with human raters in terms of Cohen’s *κ* (Table A1 in the Supplementary Material). The *κ* coefficients varied considerably between 0.59 for (R437Q07/C1, R456Q02/C1) and 0.97 (R455Q03/C1, R455Q03/C2). Within-item differences were minor, except for items R453Q04 and R456Q02, where C2 outperformed C1 substantially. Similarly, the error rates ranged from false-positive rates of up to 49.0% (R456Q02/C1) and false-negative rates of up to 43.0% (R437Q07/C1) to false-positive rates as low as 1.7% (R437Q07/C2) and false-negative rates as low as 0.1% (R455Q03/C2).

### 3.2. Method

#### 3.2.1. Ability estimation

We computed an EAP ability estimate based on the manual scores of the eight items and the 2PL item parameters from PISA scaling. This reference ability estimate (EAP reliability = .584) was used to test whether the classifier error rates included a dependency on ability and as a point estimate for ability when fitting the VER model, as described in the next section.

The automatically coded items were arranged in two forms according to the classifier used. For each of the forms, EAP ability estimates were computed using the 4PL and G4PL models with the automatically coded responses. For the 2PL and 4PL models, the PP package (Reif & Steinfeld, 2021) was used, while for the G4PL model, a rectangle-rule quadrature of the posterior distributions was employed, using 100 nodes equally spaced in the interval 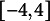 .

To evaluate the merits of our models that consider classification errors, we used two baselines: the reference ability estimate as described above and an EAP estimate based on automatic scores and the 2PL model for the manual score. The latter corresponds to an approach that ignores deviations between manual and automatic scores, using the model for the manual scores but with automatic scores.

A relatively non-informative normal prior with 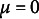 and 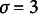 was used for all ability estimates to avoid excessive inward bias.

#### Conditional independence of classification outcome and 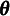


3.2.2.

We tested the conditional independence assumption (Equation (9)) using the characterization by constant classifier error rates, as defined in Equation (10). We denote 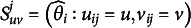 as the subsample of proficiencies of test takers whose response to item 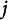 was scored as 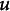 by the human rater and as 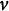 by automatic scoring. If the probability of false-negative classification does not vary with the ability level, then, according to Equation (10), the split of test takers whose manual score indicated a correct answer into 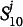 and 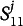 is purely random. Analogous deliberations hold for 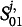 and 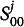 assuming a constant probability of false-positive classifications. Based on this rationale, a two-sample Kolmogorov – Smirnov test was used to test the null hypothesis that 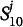 and 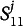 , as well as 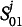 and 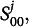 are samples from the same distributions. We tested each combination of items and classifiers for varying error rates for both error types, resulting in two tests per item. A significance level of 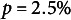 was used, applying a Bonferroni correction of the cumulative 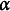 -error of 5% at the item level, that is, the probability of rejecting at least one true null hypothesis out of the two tested per item.

#### 3.2.3. Fitting the classifier error model

We fitted the CER model to all items. Using Equations (14) and (27), the maximum likelihood estimates for the classifier parameters, and hence, the third and fourth parameters of the marginal 4PL were derived from the false-positive and false-negative rates of the classifier, respectively, which are given in Table A1 in the Supplementary Material.

The VER model was used for automatically coded items for which the Kolmogorov – Smirnov test indicated nonconstant error rates. Each of the two error types, false positive and false negative, was modeled separately according to the outcome of the test. Specifically, we modeled the log odds of misclassification of the error type in question using a logistic regression model (Equations (28) and (29)) that employed the reference ability estimate as a predictor, yielding a regression model for each modeled error type.

### 3.3. Results

#### 3.3.1. Conditional independence and classifier error models

Table A2 in the Supplementary Material summarizes the results of the proposed Kolmogorov – Smirnov tests, in which, as stated above, the null hypothesis corresponds to the case of constant error rates. For false-positive classifications, the null hypothesis was rejected for all but one item, R437Q07 (both classifiers). For false-negative classification, the null hypothesis was rejected for four automatically scored items (R432Q05/C2, R456Q02/C2, R456Q06/C1, and R456Q06/C2). This suggests that for all but one item, the classification error rates varied with the level of the latent trait; hence, the assumptions of our rationale for applying the CER and 4PL models for these items were, to some extent, violated. The assumption of constant error rates for both error types was maintained for only one item: R437Q07 (C1 and C2).

**Figure 3:**
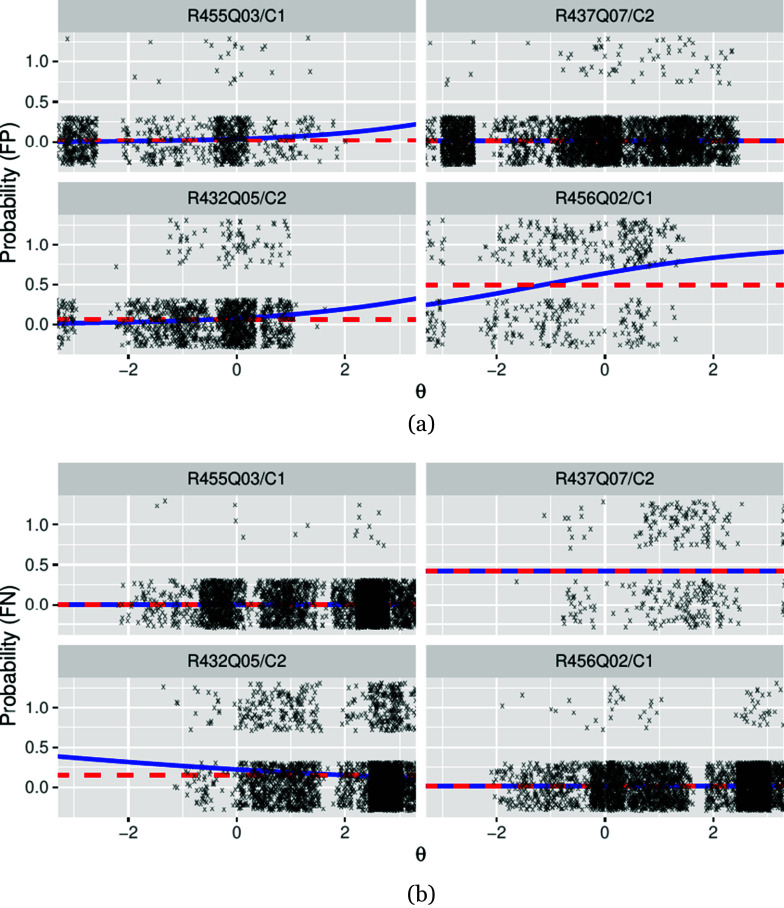
Fitted classifier error models of four exemplary items. (a): conditional probability of false-positive classification, (b): conditional probability of false-negative classification. Blue solid line: G4PL, red dashed line: 4PL. Error models for both models coincide where constant error rates were used with the G4PL in accordance with results from independence testing. Jittered data points are overlaid (a: ordinate 1—false positives, ordinate 0—true negatives, b: ordinate 1—false negatives, ordinate 0—true positives. The amount of jitter is ±.3 for both directions.

Figure 3 shows the logistic regression curves fitted to model the conditional probabilities of false positives (a) and false negatives (b). As evident in Figure 3, the data was sparse in the upper and lower 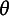 range for false positives and false negatives, respectively. This is due to the background rates of correct and incorrect responses, which also depend on item difficulty.

#### 3.3.2. Functioning of automatically coded items

We discuss the model-implied effects of automatic response coding on item characteristic curves and item information curves under the 4PL and G4PL models for four of the items. The four exemplary items were selected to cover different characteristics of the automatic scoring regarding the extent of misclassification and the use of constant or variable error rates in accordance with the results of the conditional independence tests.

**Figure 4:**
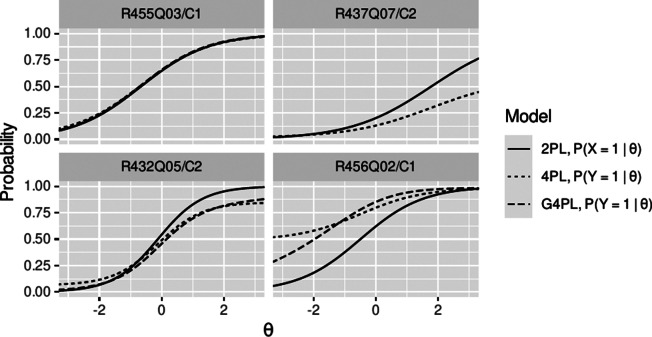
Item characteristic curves of four exemplary items. Item characteristic curves of four exemplary items, giving the probability of observing a response scored as correct by manual scoring (2PL model, solid line) and, respectively, automatic scoring (4PL model, dotted line, G4PL model, where fitted: dashed line).

**Figure 5:**
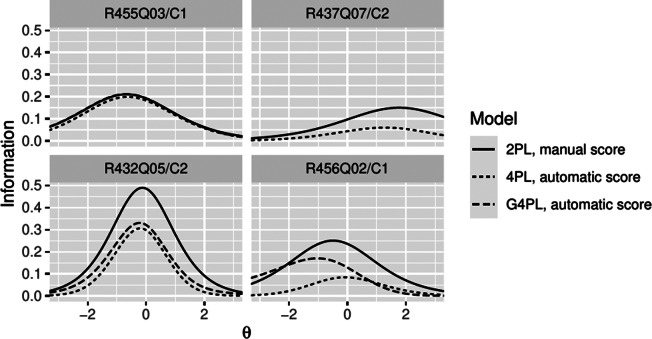
Item information curves of four exemplary items. Item information curves of four exemplary items under manual scoring (2PL model, solid line) and, respectively, automatic scoring (4PL model: dotted line, G4PL model, where fitted: dashed line).

For item R455Q03/C1, automatic scoring worked excellently (Table A1 in the Supplementary Material). Under the 4PL model, the false-positive rate of 2.3% introduced a lower asymptote at .023, and the false-positive rate of 0.5% introduced an upper asymptote at .995 in the item characteristic curve of the automatically scored item. As depicted in Figure 4 (top left), the low error rates of automatic scoring led to an item characteristic curve of the automatically scored item that was only slightly different from that of manual scoring. The rising rate of false positives with increasing 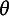 (Figure 3a, top left) modeled in the G4PL was too slight to make a significant difference to the uniform error rates in the 4PL. However, a decrease in item information was still discernible for the 4PL model (Figure 5, top left) versus the 2PL model of manual scoring.

The classification accuracy for item R437Q07/C2 was characterized by a low rate of false positives but a substantial rate of false negatives. The resulting upper asymptote at .581 dominated the effect of automatic scoring modeled by the 4PL model (Figure 4, top-right). Consequently, a loss of information relative to manual scoring was observed (Figure 5, top-right). In accordance with the results of the independence testing, the G4PL was not applied to this item.

For item R432Q05/C2, the classifier exhibited misclassification rates of 6.3% (FP) and 15.1% (FN). Hence, the impact of both asymptotes on the item characteristic curves is noticeable (4PL; Figure 4, bottom left). The variable error rates used with the G4PL model predicted that, as ability increases, the probability of false-negative classification decreases and the probability of false-positive classification increases. Consequently, the variable false-positive error rate fell below the constant (mean) error rate in the low ability range, and the variable false-negative error rate remained under the constant (mean) error rate in the high ability range (Figure 3a,b, bottom left panels). Hence, for the G4PL model, the lower asymptote was higher and the upper asymptote was lower than for the 4PL model. The ICC of the G4PL followed that of the 2PL model more closely than that of the 4PL (Figure 4, bottom left), and the model-implied loss of information incurred by automatic scoring was smaller for the G4PL than it was for the 4PL model (Figure 5, bottom left panel).

Finally, item R456Q02/C1 showed a high false-positive rate (49%) and a low false-negative rate (1.5%). Here, for the 4PL model, the lower asymptote dominated, and item information was attenuated accordingly (Figures 4 and 5, bottom right). Item R456Q06 shows that the change in item information between the 2PL model and G4PL models does not necessarily reduce across all ability levels. This is plausible because the dependence of error rates on the ability trait implies that discrepancies between scoring methods can carry information about ability. For item R456Q06/C1, this is expressed in the values of item information of the G4PL model, which were slightly higher than those of the 2PL model in the lower ability range. Error models, item characteristic curves, and information curves for the remaining items are included in Figures A2–A5 in the Supplementary Material).

**Figure 6:**
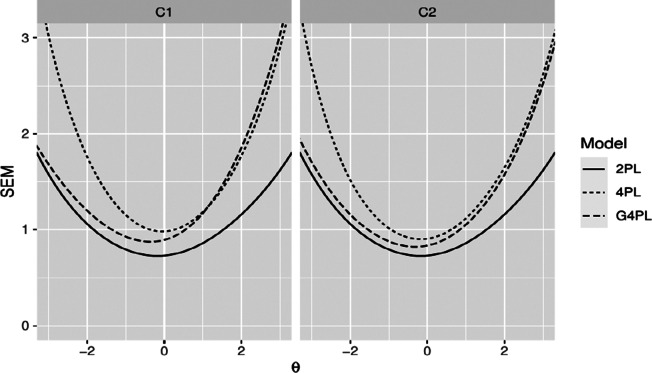
Standard error of measurement (SEM). SEM for manual scoring (2PL) and automatic scoring (4PL model: dotted line, G4PL model: dashed line) for both classifiers and test forms comprising eight PISA items.

The overall reduction in information incurred by automatic scoring also led to an increase in the standard error of measurement at the test level. This reflects the uncertainty introduced by automatic scoring. Figure 6 shows an overall increase in the standard error owing to automatic scoring for both test forms constructed from the set of eight items per classifier.

#### 3.3.3. Reliability of ability estimates obtained from automatic scores

We now consider the extent to which trait measurements obtained from human-coded responses can be reproduced by replacing them with automatically coded responses. This is a matter of reproducibility of scores by different assessments, and hence, a question of reliability. We assessed the relative reliability of the ability estimates based on their association, measured using Pearson’s correlations. In our framework, the human-coded responses are regarded as the gold standard; therefore, a high degree of association with measurements obtained from human-coded responses is desirable for any measurement obtained from automatically coded responses.

Hence, ability estimates based on manual scores and 2PL item parameters form the reference frame. To avoid data leakage, we randomly split the dataset into a training set comprising 90% of the data for each item (between 3736 and 3826 persons per item) and a test set comprising the remaining 10% (between 408 and 436 persons per item). The ability estimates reported in this section were computed for the test set using the parameters of the models fitted to the training set. Table 1 shows the correlations between the ability estimates obtained using the different models and scoring variants. For classifier C1, the ability estimates obtained using automatic scores correlated at approximately .81 to .82 with those obtained from manual scores. For classifier C2 at approximately .85, the correlations were slightly higher, consistent with the higher average agreement between C2’s scoring and the manual scoring. For both classifiers, the association between the ability estimates obtained from the automatic scores, at .96 and above, was nearly perfect, with minor differences between the models.

**Table 1:** Correlations of ability estimates (all test-takers)

Model		Manual	Automatic		
		2PL	2PL	4PL	G4PL
Manual	– 2PL	—	.820	.806	.808
Automatic	– 2PL	.848	—	.963	.974
		– 4PL	.852	.960	—	.993
		– G4PL	.849	.967	.993	—

*Note:* The correlation coefficients for classifiers C1 and C2 are presented above and below the diagonal, respectively.

From these numbers, we may assume that in the complete sample, first, the degree of relative reliability of the measurements obtained from the automatic scores is quite good, and second, the correlation coefficients being very close to each other, there seems to be no clear advantage of the proposed models for automatic scores over the baseline that ignored misclassification. However, in our dataset, the majority of the test-takers (71.9% and 76.0% for C1 and C2, respectively) did not experience any classifier errors (Table A3 in the Supplementary Material).

For this substantial fraction of cases in which the manual and automatic scores were in perfect agreement, the assumption of error-free classification, essentially made when using the 2PL model with automatic responses, holds true, leading to an advantage of this approach.

For this error-free subset of the test set (C1: N = 660, C2: N = 713), at above .97, the 4PL and G4PL estimates were highly correlated with the reference ability estimate (Table A4 in the Supplementary Material). Table 2 shows the correlations between the ability estimates for the remaining portion of the sample, namely the subsample of persons in the test set who experienced at least one misclassified response (C1: N = 283, C2: N = 230). The same pattern is observed for both classifiers. The correlations between the reference ability estimate and those obtained using the 2PL with the automatic score were markedly lower than those in the complete test set, reflecting the effect of errors in automatic scoring. The estimates obtained using the proposed 4PL and G4PL models exhibited higher correlations with the reference ability estimates. The more flexible G4PL model did not perform better in terms of relative reliability. As in the complete test set, the associations between the estimates obtained from the automatic scores were nearly perfect (.94 and above). These results indicate that, in the presence of classification errors, by using either one of the 4PL or G4PL models, relative reliability was increased over the 2PL model.

**Table 2:** Correlations of ability estimates (test-takers with one or more classification errors)

Model		Manual	Automatic		
				2PL	2PL	4PL	G4PL
Manual	– 2PL	—	.295	.385	.365
Automatic	– 2PL	.235	—	.953	.962
		– 4PL	.421	.940	—	.992
		– G4PL	.377	.955	.992	—

*Note:* Correlations for classifiers C1 and C2 are presented above and below the diagonal, respectively.

#### 3.3.4. Bias in ability estimates

For classifier C1, the ability estimates obtained from the automatic scores and the 2PL model exhibited a bias relative to the reference ability estimate of 0.183 (95% CI: [0.113, 0.254]). For classifier C2, the bias was −0.013 (95% CI: [−0.078, 0.052]). This finding of positive bias for C1 is consistent with the results from our simulation study, as C1 leaned toward higher false-positive rates (mean FPR: 20.4%, mean FNR: 9.7%), whereas for C2, false-positive and false-negative rates were nearly balanced (mean FPR: 11.5%, mean FNR: 10.4%). For the 4PL model, bias of −0.116 (95% CI: [−0.191, −0.041]) was reversed in sign to and slightly decreased in magnitude relative to the 2PL for C1, while for C2, at −0.015 (95% CI: [−0.081, 0.050]), as for the 2PL, bias was not statistically significant. Again, these findings are consistent with our simulations. For the G4PL model, however, for both C1 and C2, the bias was negative and of greater magnitude than that for the 2PL model (C1: −0.276, 95% CI: [−0.347, −0.205], C2: −0.198, 95% CI: [−0.263, −0.133]). This last result is unexpected: In our simulations, the magnitude of bias in ability estimates obtained from automatic scores using the G4PL was substantially smaller than when using the 2PL, when the error rates were unbalanced, and bias was statistically insignificant when error rates were balanced.

Overall, the results on bias in our empirical example are consistent with our simulations, except for the G4PL model, which exhibited a greater magnitude of bias than we expected based on the simulation results.

## 4. Discussion

This article addresses an essential challenge in the application of automatic scoring for open-ended test items in educational assessments based on IRT models, namely, ability estimation, which accounts for the additional uncertainty of automatic scoring.

The approach proposed here posits that the manual score and the accompanying IRT model fitted to them define the frame of reference. In this framework, automatic scores are the output of an error-prone process, and their deviation from manual scores is modeled. Our approach enables access to the information in open-ended items for immediate scoring, which is useful for providing instant reporting or feedback, or for enhancing adaptivity during testing. In applications such as PISA assessments, approaches to reduce measurement error by increasing adaptivity, such as MSAT or the highly adaptive testing (HAT; Frey et al., 2023), can be complemented and enhanced by using information in open-ended items. In these contexts, our models can be used flexibly; for instance, automatic scores can be used during online testing to inform routing decisions in an adaptive design, whereas manual scores can be supplemented to maximize the reliability of reported ability estimates. Another important feature of our approach is that it allows quantification of the loss of measurement precision due to imperfect automatic scoring in terms of item information and, by extension, the standard error of measurement. This feature has the potential to guide decisions on which classifiers to select for a particular test and population and, on an individual basis, which items should be submitted to manual scoring to reduce measurement errors cost-efficiently.

Within our framework, we proposed two models that differ in the underlying assumption of how classifier error rates relate to latent ability. The assumption of a classification error conditionally independent of ability led to a simple CER model and a marginal 4PL model for automatic scores. If the error rates were allowed to depend on ability, the VER model resulted in a marginal G4PL model for automatic scoring. The results of a simulation study demonstrated successful parameter recovery for both the CER and VER models, whereas the sample size requirements of the VER model were considerably higher.

Simulation results indicate that ability estimates computed from automatic scores using the model for manual scores can be affected by considerable bias when misclassified responses of one of the error types dominate. In the simulations, the estimates obtained using the 4PL and G4PL models remained unbiased.

In our empirical example, based on data from PISA 2012 (OECD, 2014), we found that the assumption of constant error rates was at least partially violated. Most of the automatically scored items exhibited a dependency of the classifier error rates on the ability level. By analyzing the ability estimates obtained from automatic scores, we found that in the presence of classification error, both the 4PL and G4PL models improved the relative reliability over a baseline that ignored the possibility of classification error. Consistent with our simulations, ability estimates from automatic scores exhibited a positive bias when the model for the manual score was used, and the classifier’s error rates leaned toward a higher rate of false positives. The estimates obtained from the 4PL led to a decrease in the magnitude of the bias; however, for the G4PL, contrary to expectations, the bias increased. Overall, the CER model performed better than the VER model in our empirical data, although its underlying assumptions were partially violated. This may be explained by the sensitivity of the logistic error rate models to influential observations, particularly in extreme ability ranges where the data were sparse. This issue can be addressed by imposing regularization; for example, using appropriate priors in a Bayesian framework. The weaker performance of the G4PL may also be due to undercomplex error models, which could be addressed in future studies by including additional predictors.

The present study has several limitations. A fundamental problem arises from the modeling approach itself. The decision to center around the manual scores as the reference causes the differences between the human and automatic scores to appear solely as classifier errors. This carries the risk of masking errors in manual scores, which can never be completely ruled out, for instance, due to biased raters or ambiguous scoring rubrics. It also disregards the machine’s potential capacity to avoid certain types of errors that humans invariably make, for example, because of fatigue. This limitation is inherent in our framework but could be overcome by more symmetric models that treat manual and automatic scores as equal sources of evidence. Accurate manual scoring is a prerequisite for reliable and valid inferences. In the context of automatic scoring, improving the accuracy of human scoring offers additional benefits by providing higher-quality training data for automatic scoring. To this end, a qualitative assessment of the responses with discrepancies between human and automatic scores may provide valuable insights.

The appeal of the simple 4PL model brings into focus the question of whether classifiers that exhibit error rates independent of ability level can be specifically designed. It may be possible to approach this problem by incorporating fairness constraints with respect to ability when training the classifiers (e.g., Zafar et al., 2019). However, the assumption of the VER model that error rates depend on ability implies that the automatic score contains information that complements the manual score. Because our focus was mainly on ability estimation using marginal models, this aspect remains unexplored.

Further limitations include the low number of automatically scored items in our empirical example, resulting in low reliability of the reference estimates, which were used in the estimation of the VER classifier model parameters and as a reference against which estimates from the proposed models are evaluated. Generalizability is further limited by the restriction of one content domain and language, and the choice of the two classifiers. In addition, the question of whether automatic scoring can work uniformly across many languages remains unanswered. Applications in international large-scale assessments, such as PISA, may lead to a large number of unique item parameters arising from differences in the performance of language-specific classifiers. Finally, classifier error rates may exhibit dependencies on person variables other than ability, or the dependency on ability may be explained by including other predictors such as gender or grade level in the model. From a technical perspective, such predictors can be easily included in the model.

## Supporting information

Bengs et al. supplementary material Bengs et al. supplementary material

## Data Availability

The datasets generated and/or analyzed in this study and the R (R Core Team, 2025) scripts used to generate and/or analyze the data are available at https://doi.org/10.17605/OSF.IO/M5EZS.

## Footnotes

1

https://doi.org/10.17605/OSF.IO/M5EZS

## Supplementary material

To view supplementary material for this article, please visit http://doi.org/10.1017/psy.2025.10018.
